# Enteric infection and dysfunction—A new target for *PLOS Neglected Tropical Diseases*

**DOI:** 10.1371/journal.pntd.0006906

**Published:** 2018-12-28

**Authors:** Michael B. Arndt, Judd L. Walson

**Affiliations:** 1 PATH, Seattle, Washington, United States of America; 2 Department of Global Health, University of Washington, Seattle, Washington, United States of America; 3 Department of Epidemiology, University of Washington, Seattle, Washington, United States of America; 4 Department of Medicine, University of Washington, Seattle, Washington, United States of America; 5 Department of Pediatrics, University of Washington, Seattle, Washington, United States of America; 6 Childhood Acute Illness and Nutrition Network, Nairobi, Kenya; Massachusetts General Hospital, UNITED STATES

The past decade has seen a dramatic increase in global attention to neglected tropical diseases, with remarkable momentum in policy, programs, funding, and research. As we look to the future, it is critical for *PLOS Neglected Tropical Diseases* to continue to reevaluate the scope of the journal and to define which syndromes and diseases continue to disproportionately impact neglected populations in low-resource settings.

Many pathogens highlighted in *PLOS Neglected Tropical Diseases* have profound impacts on the gastrointestinal system. There is increasing recognition of the role of the intestine as the critical site where the human host encounters pathogens, initiates the immune response, protects from pathogen invasion, controls nutrient and calorie absorption, and modulates hormonal response. Enteric dysfunction—as a result of diarrheal disease, parasitic infections, alterations in the gut microbial environment, and other causes—has been linked to malnutrition, delayed cognitive development, increased susceptibility to other infectious diseases, poor oral vaccine response, and increased mortality risk. The collective long-term impacts of these conditions on the most marginalized communities are tremendous. Given the importance of these diseases and syndromes, *PLOS Neglected Tropical Diseases* has decided to include a new section focused on enteric infections and enteric dysfunction. Of course, many diseases and syndromes—including the soil-transmitted helminths, schistosomiasis, giardiasis, cholera, and other enteric pathogens that impact the physiology of the gut—are already included within the scope of *PLOS Neglected Tropical Diseases* and have been highlighted previously in this journal [1]. It is our hope that by highlighting the shared impacts of enteric infection and dysfunction as a specific target for the journal, we can serve as a major platform to bring together valuable discoveries and innovations that will drive progress in this important field.

Diarrhea remains a leading cause of child mortality globally. The Global Enteric Multicenter Study (GEMS) reported *Shigella* spp., rotavirus, adenovirus, heat-stable toxin enterotoxigenic *Escherichia coli* (ST-ETEC), *Cryptosporidium* spp., and *Campylobacter* spp. as the most common pathogens associated with moderate-to-severe diarrhea in infants and young children in low-resource settings [2, 3]. However, mounting evidence also suggests that infection with enteric pathogens need not cause diarrhea to pose threats to child health. Even in the absence of diarrhea, many pathogens can increase gut inflammation and permeability and may result in systemic inflammation [4]. For example, high *Campylobacter* pathogen burden was associated with a significant decrease in height for age z-score among children enrolled in a multisite birth cohort study (MAL-ED), independent of diarrhea symptoms [5]. In addition to negative growth impacts, there is mounting evidence that frequent enteric infections and poor gut health in infants and children may also impair cognitive development and increase risk of metabolic and cardiovascular diseases later in life [6–8]. Although innovations such as low-osmolarity oral rehydration solution and zinc have greatly reduced the acute mortality from diarrhea in low-resource settings, interventions to reduce these long-term sequelae associated with repeated symptomatic and asymptomatic exposure to enteric pathogens are limited.

In many low-resource environments, environmental enteric dysfunction (EED)—a small bowel intestinal disorder characterized by mucosal inflammation, reduced barrier integrity, and nutrient malabsorption (e.g., villus blunting)—is widespread among infants and children, including children with no other identified comorbidity [9–11]. Noninvasive biomarkers reflective of intestinal inflammation, intestinal permeability, and microbial translocation appear to be elevated among more than half of all children in many settings. In addition to direct effects within the gut, EED can lead to translocation of bacterial products across the compromised intestinal barrier, with resulting systemic inflammation. Such inflammation appears to interfere with the growth-hormone (GH) axis and leads to a state of GH resistance that ultimately suppresses growth. GH resistance, defined by elevated systemic GH levels and low levels of insulin-like growth factor 1 (IGF-1; the hormone principally responsible for stimulating local bone and tissue growth), is exhibited in states of caloric and protein undernutrition, isolated micronutrient deficiencies (zinc, vitamin A, magnesium), and in response to chronic systemic inflammation [12–16]. Evidence for the relationships between EED, systemic inflammation, and GH resistance in children comes from studies of young children in Brazil and Zimbabwe in which blood-based biomarkers of systemic inflammation (e.g., C-reactive protein and Alpha-1-acid glycoprotein) were negatively associated with systemic IGF-1 (and IGF binding protein 3) and linear growth, and in Brazil, positively associated with stool myeloperoxidase, a marker of intestinal inflammation [13, 16].

However, despite the highly prevalent nature of EED and the associated morbidity and mortality risk, there are major gaps in our understanding of EED and enteric dysfunction across the entire spectrum of translational research. *PLOS Neglected Tropical Diseases* is uniquely positioned to highlight research addressing these gaps from basic research to the implementation of interventions at scale.

Because the etiology and physiology of enteric dysfunction is incompletely defined, it is essential that model systems (e.g., animal models, organoids, in vitro cell models) be developed to elucidate some of the underlying features of the condition. Few published studies have evaluated the histopathologic features of EED. One such study produced a mouse model of EED using a combination of protein-deficient diet and exposure to a specific microbial environment [17]. Ongoing work using this model is exploring the impact of EED histopathology on brain development, both through direct (dissection) and indirect (behavioral) methods. Further work must be done to determine whether this model is sufficiently robust to serve as a preclinical model for EED interventions and/or oral vaccine efficacy in low-resource settings and to screen clinical candidates prior to use in phase 1 studies. It also remains unclear to what extent changes in the gut are protective and appropriate rather than destructive and harmful to the health of the host. To date, limited research has explored the potential role of host genetics in inflammatory, metabolic, and immune pathways involved in enteric dysfunction. In addition, it is not clear whether there is interplay between the composition and activity of the gut microbiome and the severity of morbidity experienced by children with EED. Despite evidence suggesting the importance of the microbiome in the normal development of endocrine, digestive, and immune systems, the optimal microbial composition of the gut remains unclear [18]. Identification of therapeutic or preventative targets for EED could draw heavily upon the insights derived from research into these areas.

There remain major challenges in establishing formal case definition(s) for enteric dysfunction that can be used in clinical trials and surveillance studies. The lack of well-validated biomarkers suitable for use in low-resource settings and predictive of morbid sequelae limits the ability of clinicians to diagnose and manage EED. The optimal method to assess gut abnormalities is endoscopy with biopsy to determine villus blunting and other histopathologic features. However, these invasive procedures may not be practical or ethical to utilize among children in low-resource settings. Because several forms of enteric dysfunction are often coendemic in low-resource settings, it is critical to harmonize case definitions to ensure proper classification of similar clinical entities from differing etiologies, because the etiology is likely relevant for elicitation of response to a given treatment approach. A combination of anthropometric, intestinal biomarker, and systemic biomarker information could potentially be used as the foundation for a case definition for EED [19]; however, more data are needed to form consensus around the ideal criteria. A case definition may also be helpful in focusing field and laboratory research on outcomes of enteric infections other than acute diarrhea, such as linear growth faltering, impairment in cognitive development, and later-life cardiovascular and metabolic disease [6]. Outside of clinical research, a case definition would provide information on the geographic distribution of EED and could enable the identification of potential risk factors that cluster geospatially. Such a case definition would also be instrumental in quantifying the long-term societal and economic impacts of EED (and chronic enteropathogen infections) in low-resource settings. An improved understanding of the epidemiology of EED will be useful in establishing whether EED is a syndrome or a disease and will be useful in guiding the approach to evaluating treatment and/or prevention interventions.

Many candidate biomarkers that can be analyzed noninvasively from stool, blood, and urine samples have been evaluated, and several have been associated with linear growth and other health outcomes [20]. Noninvasive markers indicative of intestinal inflammation (fecal neopterin, myeloperoxidase, and calprotectin), barrier disruption (fecal alpha-1-antitrypsin), and intestinal damage and repair (serum intestinal fatty acid binding protein, serum glucagon-like peptide 2, and fecal regenerating family member 1- beta [Reg1β]) [21–24] have been associated with reduced growth and adverse health outcomes. The dual sugar permeability test, which measures the ratio of urinary lactulose excretion to rhamnose (or mannitol) excretion, following fasting and saccharide administration, is a measure of intestinal barrier function that has long been utilized as a biomarker for EED [25, 26]. Numerous blood-based biomarkers of systemic inflammation have been correlated with linear growth faltering and/or oral vaccine failure, including acute phase proteins (i.e., alpha-1-glycoprotein, ferritin, and C-reactive protein), innate immune response (soluble cluster of differentiation [CD]14), indoleamine 2, 3-dioxygenase 1 (IDO1) activity (kynurenine and tryptophan), and cytokines (e.g., interleukin [IL]-1b and IL-10) [4, 27–29]. However, relationships between these markers and health outcomes have not been observed consistently across studies conducted in different geographies and age groups, and this heterogeneity is not yet well understood [30]. It is unclear whether there are important differences in etiology underlying enteric dysfunction in each setting, and although some studies have sought to combine information from different markers into composite scores [22, 31, 32], methods and markers have differed. These fecal, urinary, and systemic biomarkers nonetheless represent potential tools for diagnosing EED and stratifying child populations with increased morbidity risk into clinical trials with therapeutic candidates.

A number of interventional approaches for enteric dysfunction have been tested in children over the past 10 to 15 years, including water, sanitation, and hygiene (WASH) interventions; zinc; antibiotics; probiotics; and the targeted administration of locally active anti-inflammatory drugs. Most of these studies have used the dual sugar permeability test as an outcome measure, with many focusing on lactulose excretion alone (rather than the ratio with mannitol or rhamnose) due to its reduced complexity. Neither a probiotic (*Lactobacillus rhamnosus* GG) nor antibiotic (rifaximin) reduced lactulose excretion in children living in Malawi [33, 34]. Although an initial study observed a significant reduction in the change in lactulose:mannitol (L:M) ratio in Malawian children receiving albendazole or zinc tablets, a follow-up study did not observe reduced L:M ratio or lactulose excretion after 12 to 24 weeks in children given a combination of zinc, albendazole, and multiple micronutrients in comparison with children given a placebo [35, 36]. A study in 3-to-9-month-old Gambian infants also did not observe a reduction in lactulose excretion or intestinal inflammation (fecal calprotectin levels) in children supplemented with long-chain polyunsaturated fatty acids (fish oil) [37]. A small pilot study in young Kenyan children with severe acute malnutrition and stunting concluded that mesalamine was safe to administer in the population and observed modest reductions in markers of intestinal inflammation (fecal calprotectin) and systemic inflammation (plasma Immunoglobulin G to endotoxin core antibody) in the treatment arm but reported better nutritional recovery in the placebo arm [38]. An upcoming publication will report on the results of a clinical trial in Malawi that tested the effect of lactoferrin and lysozyme administration on intestinal integrity and growth in infants [39]. Results from two major cluster-randomized studies of WASH interventions, the WASH Benefits trial (Bangladesh and Kenya) and SHINE (Zimbabwe), examined whether such interventions impact growth, gut integrity, or inflammation. Neither trial has reported improvements in child linear growth, diarrhea, or cognitive scores in individual water, sanitation, hygiene, or combined WASH arms compared with their control groups [40, 41], and secondary analyses of other outcomes are currently being analyzed or are in press. Plausible explanations for the lack of health improvements in these WASH trials come from analyses of environmental samples collected in Kenya that suggest the great importance of child contact with surface water, soil, and public surfaces as sources of enteric pathogen exposure [42, 43]. Additional innovation(s) may be necessary to effectively alter the pertinent behavioral, environmental, and spatial conditions to disrupt enteric pathogen exposure and prevent associated health outcomes.

*PLOS Neglected Tropical Diseases* looks forward to publishing the results of studies and trials targeting the definition, classification, mechanisms, treatment, and/or prevention of enteric infection and dysfunction. *PLOS Neglected Tropical Diseases* is prepared to serve as a platform to bring together and disseminate important discoveries and innovations that will drive solutions to these enteric conditions that disproportionately impact populations in low-resource settings.
